# Probiotic Bacteria Produce Conjugated Linoleic Acid Locally in the Gut That Targets Macrophage PPAR γ to Suppress Colitis

**DOI:** 10.1371/journal.pone.0031238

**Published:** 2012-02-21

**Authors:** Josep Bassaganya-Riera, Monica Viladomiu, Mireia Pedragosa, Claudio De Simone, Adria Carbo, Rustem Shaykhutdinov, Christian Jobin, Janelle C. Arthur, Benjamin A. Corl, Hans Vogel, Martin Storr, Raquel Hontecillas

**Affiliations:** 1 Nutritional Immunology and Molecular Medicine Laboratory, Center for Modeling Immunity to Enteric Pathogens, Virginia Bioinformatics Institute, Virginia Tech, Blacksburg, Virginia, United States of America; 2 Experimental Medicine, L'Aquila University, L'Aquila, Italy; 3 Department of Medicine, Division of Gastroenterology and Hepatology and Center for Gastrointestinal Biology and Disease, University of North Carolina at Chapel Hill, Chapel Hill, North Carolina, United States of America; 4 Department of Biological Sciences, Metabolomics Research Centre, and Department of Medicine, Division of Gastroenterology, University of Calgary, Alberta, Canada; 5 Department of Medicine, Division of Gastroenterology, University of Munich, Munich, Germany; 6 Department of Dairy Science, Virginia Tech, Blacksburg, Virginia, United States of America; Charité, Campus Benjamin Franklin, Germany

## Abstract

**Background:**

Inflammatory bowel disease (IBD) therapies are modestly successful and associated with significant side effects. Thus, the investigation of novel approaches to prevent colitis is important. Probiotic bacteria can produce immunoregulatory metabolites *in vitro* such as conjugated linoleic acid (CLA), a polyunsaturated fatty acid with potent anti-inflammatory effects. This study aimed to investigate the cellular and molecular mechanisms underlying the anti-inflammatory efficacy of probiotic bacteria using a mouse model of colitis.

**Methodology/Principal Findings:**

The immune modulatory mechanisms of VSL#3 probiotic bacteria and CLA were investigated in a mouse model of DSS colitis. Colonic specimens were collected for histopathology, gene expression and flow cytometry analyses. Immune cell subsets in the mesenteric lymph nodes (MLN), spleen, blood and colonic lamina propria cells were phenotypically and functionally characterized. Fecal samples and colonic contents were collected to determine the effect of VSL#3 and CLA on gut microbial diversity and CLA production. CLA and VSL#3 treatment ameliorated colitis and decreased colonic bacterial diversity, a finding that correlated with decreased gut pathology. Colonic CLA concentrations were increased in response to probiotic bacterial treatment, but without systemic distribution in blood. VSL#3 and CLA decreased macrophage accumulation in the MLN of mice with DSS colitis. The loss of PPAR γ in myeloid cells abrogated the protective effect of probiotic bacteria and CLA in mice with DSS colitis.

**Conclusions/Significance:**

Probiotic bacteria modulate gut microbial diversity and favor local production of CLA in the colon that targets myeloid cell PPAR γ to suppress colitis.

## Introduction

Inflammatory bowel disease (IBD) is an immune-mediated illness of unknown cause characterized by a chronic and uncontrolled inflammation of the intestinal mucosa [1]. Its two main clinical manifestations are Crohn's disease (CD) and ulcerative colitis (UC). Current therapies ameliorate IBD by inducing and maintaining clinical remission, but cannot be considered for the long-term management of the disease due to their significant adverse side effects [2]. Thus, exploring novel therapeutic and preventive approaches for IBD is novel and important.

The adult human gut contains about 100 trillion microbial organisms [3]. Changes in the composition of the gut microbiome can modulate the induction of regulatory versus effector immune responses. Therefore, manipulating the gut bacterial composition and local metabolite production by using probiotic bacteria has been explored as a promising avenue for therapeutic intervention against IBD. However, the mechanisms of action underlying the immunoregulatory effects of probiotics and their key metabolites in the gut mucosa are incompletely understood. Gut microbial populations can synthesize a wide range of lipid molecules that vary in chemical structure from short chain fatty acids (SCFA) such as butyrate, acetate and propionate to polyunsaturated fatty acids (PUFA) involved in regulating apoptosis and the immune response [2], [4], [5], [6], including conjugated linoleic acid (CLA) and conjugated linolenic acid (CLNA) isomers [7], [8]. Interestingly, CLA, punicic acid and eleostearic acid have received some attention in the treatment of colitis and colitis-associated cancer [2], [9], [10], [11], [12], [13], [14].

*Bifidobacterium breve* strains, a *B. bifidum* strain and a *B. pseudolongum* strain, produce CLA and CLNA isomers *in vitro* from linoleic acid or alpha-linoleic acid, respectively [15]. However, there is no *in vivo* evidence of production of CLA by probiotic bacteria in the gut. CLA-producing bacterial strains are found in a probiotic mixture known as VSL#3.

VSL#3 has demonstrates efficacy in patients with ulcerative colitis [16], [17], [18] and pouchitis [19], and in animal models of colitis [20]. VSL#3 probiotic bacteria are claimed to regulate intestinal microbial balance by synthesizing antibacterial substances like lantibiotics [21], and other bacteriocins-like compounds [22], competing with pathogens by preventing their adherence to intestinal epithelial cells [23], [24], suppressing gut inflammation by up-regulating anti-inflammatory cytokines, like IL-10 [25], favoring an expansion of mucosal regulatory cells during ileal pouchitis [26], and down-regulating LPS-driven production of IL-8, TNF-α and IFN-γ [27]. In contrast the the reported anti-inflammatory actions, a recent study demonstrates that pre-treatment with VSL#3 of mice in a SAMP ileitis model prevents intestinal inflammation through a mechanism involving stimulation of epithelial TNF-α, activation of NF-κB and restitution of normal barrier function [28], thereby suggesting immunostimulatory effects at the epithelial layer. Therefore, there is a lack of comprehensive understanding of the mechanisms of action underlying the protective effects of probiotics. This study aims to investigate the mechanisms of immunoregulation of gut probiotic bacteria in mice by focusing on their ability to produce anti-inflammatory metabolites and influence mucosal immune responses.

## Materials and Methods

### Animal procedures and experimental diets

C57BL6 mice were used for DSS colitis (n = 60) studies. In a follow up study, we also used macrophage-specific PPAR γ conditional knockout mice with a Cre recombinase targeted to the LysM-Cre promoter (LysM-Cre+) and control LysM-Cre− (wild-type phenotype) mice in a C57BL/6J background (n = 60). For each experiment, mice were fed purified AIN-93G rodent diets (Table S1) with or without 1% CLA for 24 days prior the induction of colitis (n = 60). The CLA supplement administered orally contained a 50∶50 mixture of the cis-9, trans-11 CLA and trans-10, cis-12 isomers (Clarinol, Loders Croklaan BV). All experimental procedures were approved by the Institutional Animal Care and Use Committee.

### Ethics Statement

All experimental procedures were approved by the Virginia Tech Institutional Animal Care and Use Committee (IACUC) and met or exceeded requirements of the Public Health Service/National Institutes of Health and the Animal Welfare Act.

### Oral treatment with probiotic bacteria

Mice received 0.5 mL of the VSL#3 probiotic solution daily by orogastric gavage using a ball tip gavage needle. The probiotic solution was freshly prepared daily in sterile conditions with a final concentration of 0,0072 g VSL#3/mL, corresponding to 1.2×10^9^ bacteria per mouse/day, in phosphate buffered saline (PBS) at pH 7.1. VSL#3 is a commercial mixture composed of four strains of lactobacilli (*Lactobacillus casei*, *L. plantarum*, *L. bulgaricus*, and *L. acidophilus*), three strains of bifidobacteria (*Bifidobacterium longum, B. breve, and B. infantis*) and *Streptococcus thermophilus*. This dose was selected because probiotic concentrations ranging between 10^8^ and 10^9^ cfu/mouse/day are sufficient to efficiently colonize the intestinal mucosa of rodent [28], [29]. Further, this dose is biologically relevant since it is based on a daily intake of about 3,600 billion bacteria for an adult human weighing 70 kg.

### DSS challenge for assessment of colitis and colorectal cancer induction

To induce colitis, mice were challenged with 2.5% dextran sodium sulfate (DSS), 36,000–44,000 molecular weight (ICN Biomedicals, Aurora, OH) in the drinking water for 7 days. Disease activity indices and rectal bleeding scores were calculated using a modification of a previously published compounded score [30]. Mice were euthanized on day 7 of the DSS challenge.

### Histopathology

Colonic sections were fixed in 10% buffered neutral formalin, later embedded in paraffin, and then sectioned (6 µm) and stained with H&E for histological examination. Tissue slides were examined in an Olympus microscope (Olympus America Inc., Dulles, VA). Colons were scored for leukocyte infiltration, epithelial erosion and mucosal thickness.

### RNA isolation and real-time polymerase chain reaction of cytokines

Total RNA from colon was isolated using the Qiagen RNA isolation kit (Qiagen) according to the manufacturer's instructions, and then was used to generate the cDNA template using the iScript cDNA synthesis kit (Bio-Rad, Hercules, CA) and real-time RT-PCR was performed as previously described [2].

### Immunophenotyping

Spleen and mesenteric lymph nodes (MLN) were excised and single-cell suspensions of tissues were resuspended in PBS and enumerated with the Coulter Counter (Beckman Coulter, Fullerton, CA). Colon samples were processed for lamina propria lymphocyte (LPL) isolation. Specifically, cells (3×10^6^ cells/well) were seeded into 96 well-plates, centrifuged at 4°C at 3000 rpm for 4 minutes, and washed with PBS containing 5% serum and 0,09% sodium azide (FACS buffer). Cells were then incubated for macrophage assessment with fluorochrome-conjugated primary antibodies to T cell and macrophage markers.

### DNA extraction, T-RFLP and community composition from feces analysis

Fecal samples from between 50–200 mg were resuspended in lysis buffer containing 20 mg/mL lysozyme and incubated for 30 minutes at 37°C to extract the DNA. Proteinase K to 350 µg/mL and 10% Sodium dodecyl sulfate was added for further lysis. Samples were homogenized using a bead beater and 0.1 mm zieconium beads (BioSpec Products, Bartlesville, OK), and then processed using a DNA extraction kit (DNeasy; Qiagen, Chatsworth, CA). The DNA extracted from each sample was used to amplify the 16S ribosomal RNA gene by polymerase chain reaction (PCR) using fluorescently labeled primers (forward primer 8F FAM 5′-AGAGTTTGATCCTGGCTCAG-3′ and reverse primer 1492R Hex 5′-GGTTACCTTGTTACGACTT-3′). Amplification products were purified using a Qiagen purification kit, and digested with HhaI, RsaI and MspI restriction enzymes to generate terminal restriction fragments (TRFs) of varying size. All data shown are from HhaI digested samples; results from RsaI and MspI digested samples gave equivalent results. The TRFs were then processed by capillary electrophoresis on the ABI 3100 genetic analyzer and size, area, and height were obtained. The size of each TRF corresponds to a different bacterium or bacterial group due to polymorphisms in the 16S rRNA gene. Size and abundance data were obtained from Genemapper and compiled into a data matrix using Sequentix software (Sequentix, Germany). These data were standardized (individual TRF peak height as a proportion of total TRF peak heights within that sample), transformed by square root, and compiled into a Bray Curtis similarity matrix using PRIMER v. 6 (Primer-e, Ivybridge, UK). To test for differences in global community composition, TRF data were subjected to hierarchical cluster followed by analysis of similarity (ANOSIM). Biodiversity of each simple was assessed by Margalef's test for richness and Shannon-Weiner diversity index; groups were compared by ANOVA followed by Tukey's test. Similarity percentages (SIMPER) were used to determine which TRFs contribute most to similarity or dissimilarity within a group.

### Fatty acid analysis from plasma samples and colonic contents

Plasma and colonic contents fatty acid methyl esters were analyzed by gas chromatography (Agilent 6890 N GC) using a CP-Sil 88 capillary column (100 m×0.25 mm i.d. with 0.2 µm thickness; Varian, Inc., Palo Alto, CA). The conditions were as follows: the oven temperature was initially set at 70°C, then increased at 8°C/min to 110°C, then increased at 5°C/min to 170°C and held for 10 min, then increased 4°C/min to 225°C and held for 15 min. The inlet and flame-ionization detector temperatures were 250°C, the split ratio was 100∶1, and a 1 µL injection volume was used. Hydrogen was used as the carrier gas and constantly flowed through the column at 1 mL/min. The hydrogen gas flow to the detector was 25 mL/min, airflow was 400 mL/min, and the flow of nitrogen makeup gas was 40 mL/min. Fatty acid peaks were identified by using pure methyl ester standards (Nu-Check Prep Inc.).

### Statistical analysis

To determine the statistical significance of the model, analysis of variance (ANOVA) was performed using the general linear model procedure of Statistical Analysis Software (SAS), and probability value (*P*)<0.05 was considered to be significant. When the model was significant, ANOVA was followed by Fisher's Protected Least Significant Difference multiple comparison method.

## Results

### VSL#3 and CLA ameliorate disease activity, gross pathology and histopathology, and colonic gene expression in mice with DSS colitis

**Figure 1:**
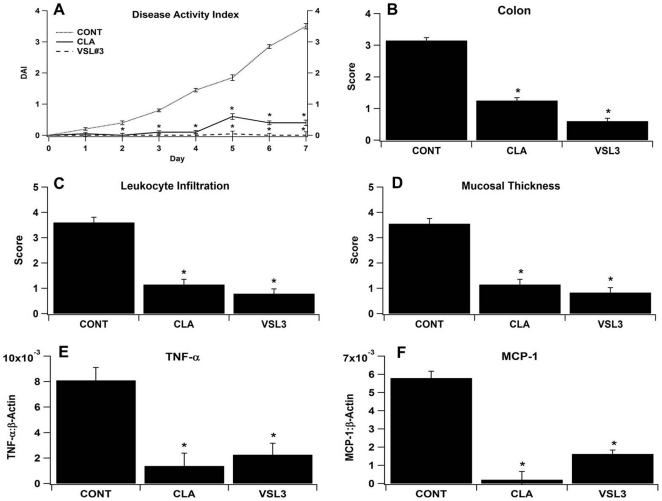
VSL#3 and conjugated linoleic acid (CLA) ameliorate disease activity. (A), colonic gross pathology (B), leukocyte infiltration (C), mucosal thickening (D), and down-modulate colonic expression of tumor necrosis factor (TNF-α, E) and monocyte chemoattractant protein 1 (MCP-1, F) in mice with DSS colitis. Data are represented as mean ± standard error. Statistically significant differences (*P*<0.05) when compared to control (CONT) mice are depicted with an asterisk.

VSL#3 and CLA treatment decreased disease activity scores associated with DSS colitis dramatically in comparison to control mice (Figure 1A). Overall, VSL#3 probiotic bacteria treatment was more effective than CLA in decreasing inflammation and reducing disease activity (Figure 1A). In line with these clinical findings, VSL#3 and CLA significantly ameliorated gross pathology in colon (Figure 1B), MLN and spleen (data not shown), colonic histopathology such as leukocyte infiltration (Figure 1C) and mucosal thickening (Figure 1D), and colonic mRNA expression such as TNF-α (Figure 1E) and MCP-1 (Figure 1F) in comparison to untreated control mice with DSS colitis.

### The loss of PPAR γ in macrophages abrogates the protective effect of VSL#3 in DSS colitis

**Figure 2:**
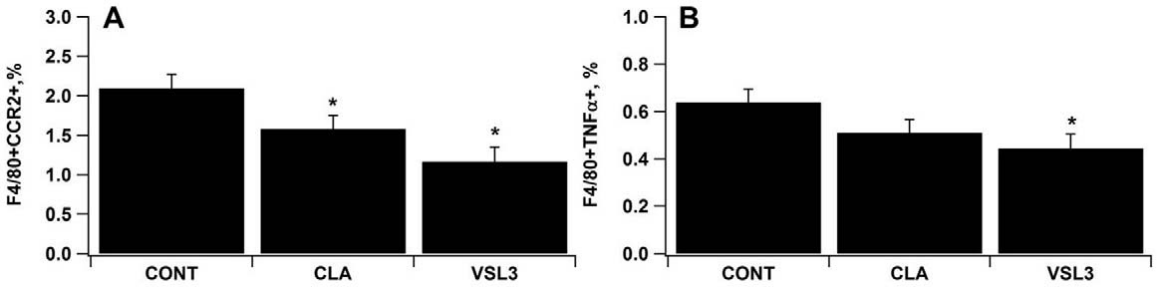
VSL#3 and conjugated linoleic acid (CLA) modulate immune cell subsets in mesenteric lymph nodes from wild type mice were immunophenotyped to identify CCR2+ (A) and TNF-α+ (B) macrophage subsets by flow cytometry. Data are represented as mean ± standard error. Statistically significant differences (*P*<0.05) when compared to control (CONT) mice are depicted with an asterisk.

**Figure 3:**
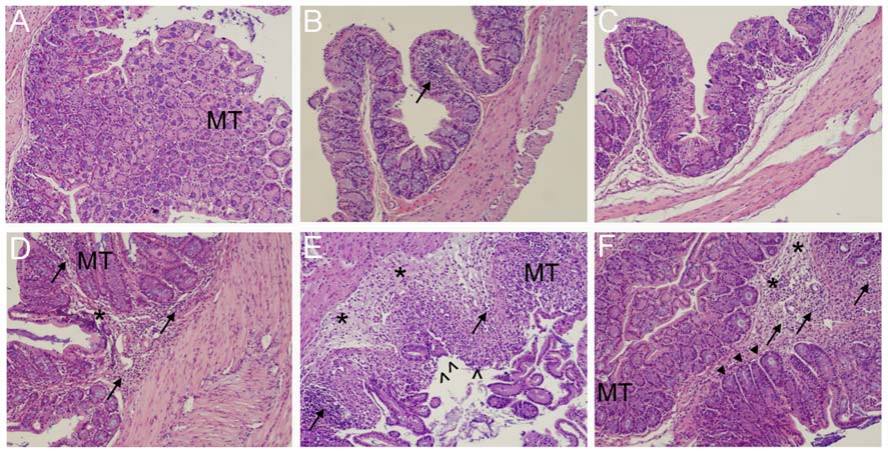
VSL#3 and conjugated linoleic acid (CLA) ameliorate colonic histopathology of PPAR γ-expressing but not macrophage-specific PPAR γ-null mice. Representative photomicrographs of colons from PPAR γ-expressing (PPAR γ flfl Cre−, top panels A–C) and macrophage-specific PPAR γ null (PPAR γ flfl Cre+, bottom panels D–F) control untreated (A&D), CLA-treated (B&E), and VSL#3-treated (C&F) mice. The colonic sections were excised on day 7 of DSS challenge, stored in formalin, sectioned and stained with hematoxylin and eosin staining. Panels B and C, corresponding to PPAR γ-expressing mice treated with CLA and VSL#3, show improved colitis. The beneficial effects of CLA and VSL#3 are abrogated in colons from mice conditionally deficient in macrophage PPAR γ (E&F). Arrows indicate leukocytic infiltration, asterisks indicate edema, filled arrowheads indicate crypt hyperplasia, open arrowheads indicate epithelial erosion and ulceration, and MT denotes mucosa thickenning. Original magnification at 100×.

**Figure 4:**
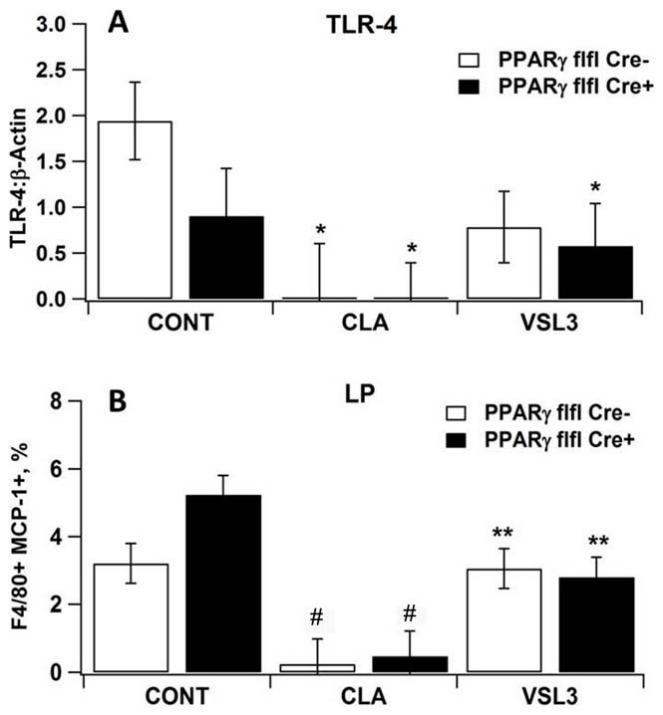
VSL#3 and conjugated linoleic acid (CLA) regulate colonic lamina propria lymphocytes (LPL) and colonic gene expression. Expression of toll-like receptor-4 (TLR-4) was assessed by quantitative real time RT-PCR (A). LP macrophages were immunophenotyped to identify their production of MCP-1 by intracellular staining and flow cytometry (B). Values are means ± SEM, n = 10. Statistically significant differences (*P*<0.05) when compared to Cre−, Cre+, or both control (CONT) mice are depicted with an asterisk, two asterisks, or the # symbol, respectively.

The percentages of CCR2 and TNF-α expressing macrophages in MLN were lower in CLA and VSL#3 treated mice with DSS colitis in comparison to the control group (Figure 2 A&B). To further characterize the role of macrophages and PPAR γ on the regulation of DSS colitis in mice, we designed a follow up experiment using a loss-of-function approach (i.e., conditional PPAR γ knockout mice). Our data indicate that the protective effect of VSL#3 in clinical disease and gross pathology was abrogated in macrophage-specific PPAR γ null mice (PPAR γ flfl Cre+) with DSS colitis. Histopathological examination of colons recovered from control PPAR γ-expressing mice (PPAR γ flfl Cre−) with DSS colitis revealed increased mucosal thickness and leukocytic infiltration (Figure 3A) when compared to CLA or VSL#3-treated mice (Figure 3 B&C). Conversely, treatment with CLA or VSL#3 did not protect macrophage-specific PPAR γ null mice from multifocal leukocytic infiltration and thickening of the colonic mucosa following DSS challenge, although it protected PPAR γ flfl Cre− mice (Figure 3 D–F). At the molecular level, VSL#3 decreased TLR-4 levels when compared to the untreated control PPAR γ-expressing mice (Figure 4A). VSL#3 probiotic bacteria and CLA reduced the numbers of MCP-1-expressing LP macrophages, regardless of the mouse genotype compared with the untreated control group (Figure 4B).

### VSL#3 and CLA decrease gut microbial diversity in a manner consistent with improved histological correlates

**Figure 5:**
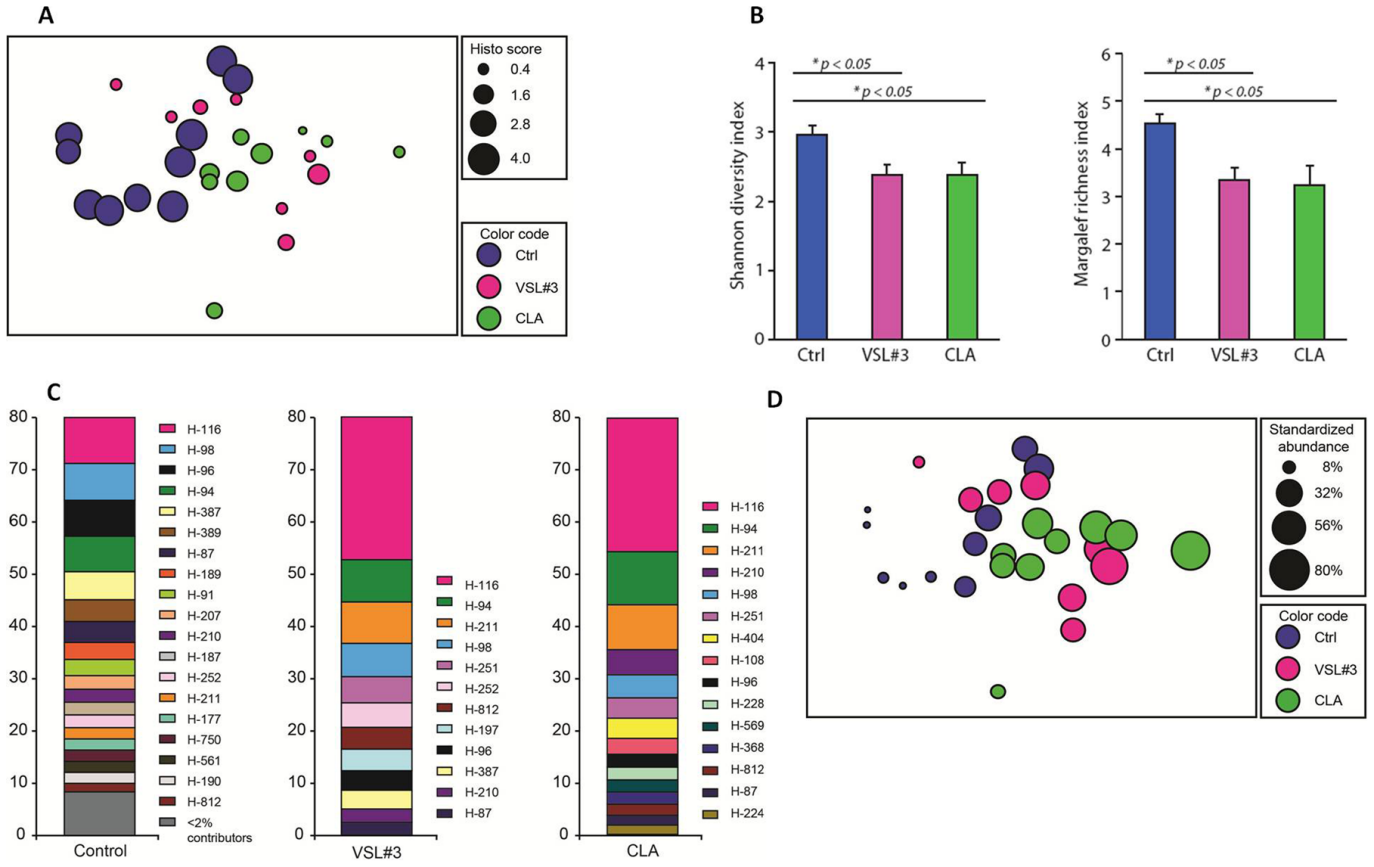
VSL#3 and conjugated linoleic acid (CLA) alter the composition of the colonic microbial community. Terminal restriction fragment (TRF) length polymorphism reveals that VSL#3 and CLA treatment alters luminal microbial community composition (A). Ordination plot by multidimensional scaling of luminal microbial communities, with circle size depicting histology score (B). Shannon diversity and Margalef richness (mean +/− SEM); groups were compared by ANOVA plus Tukey's test (C). SIMPER analysis; percent contribution of TRFs (top 80%) that contribute most to similarity within each group (D). Ordination plot in panel A, with circle size depicting standardized abundance of TRF H116.

The fecal microbiota of control, CLA and VSL#3 treated mice was assessed daily 30 days after feeding (day 0 of DSS) using terminal restriction fragment length polymorphism (TRFLP) analysis. Microbial community composition was compared between the treatment groups using Analysis of similarities (ANOSIM). Depicted in multidimensional scaling plots (Figure 5A), ANOSIM revealed the fecal microflora composition from CLA- and VSL#3-treated mice differed significantly from that of control mice (Figure 5A). More specifically, for ANOSIM comparisons: ctrl vs. VSL#3, R = 0.340, *P*<0.001; ctrl vs. CLA, R = 0.378, *P*<0.005; CLA vs. VSL#3, no difference, R = 0.087, *P* = 0.112. Moreover, microbial communities clustered by day 7 DSS histology score (circle size) (Figure 5A). To directly assess microbial diversity within each community, we calculated Margalef's richness and Shannon-Weiner diversity indices for each sample. VSL#3 and CLA treated mice exhibited significantly lower microbial diversity compared to control fed mice (VSL#3 or CLA vs. control, *P*<0.05). We used Similarity Percentage analysis (SIMPER) to determine which bacterial groups, represented as terminal restriction fragments (TRFs), best define a particular treatment. Interestingly, VSL#3 and CLA treated mice shared an enrichment and predominance of TRF H-116 (Figure 5C), which was significantly less abundant in control mice (VSL#3 or CLA vs. control, *P*<0.05 by t test). Additionally, there was a negative correlation between the abundance of this bacterial group and histology score (Spearman R = −0.525, *P*<0.005, Figure 5D).

### Plasma metabolomic analyses

Metabolic changes in plasma of control, CLA and VSL#3 treated mice induced by the DSS challenge were established using an O-PLS-DA strategy, comparing ^1^H NMR profiled metabolite concentrations obtained from DSS challenge and no challenge mice. Three O-PLS-DA models were built for control, CLA and VSL#3-treated mice. The model summary statistics, explained variance of *X* (R^2^X), DSS challenge status class variation (R^2^Y) together with cross-validated predictive abilities (Q^2^Y) are presented in Table S2. A clear discrimination was achieved between metabolic profiles of DSS challenge and no challenge mice fed control diet, as evidenced by high Q^2^Y value. Discrimination between two groups of mice with and without DSS colitis and treated with CLA was not so clear, and almost no difference was found in metabolic profiles of plasma samples from mice treated with VSL#3 (Figure S1). The degree of differential abundance of individual metabolites is also summarized in Table S2 for the predictive component distinguishing DSS challenge samples from no challenge samples.

### VSL#3 treatment increases CLA concentrations in the colonic lumen

**Figure 6:**
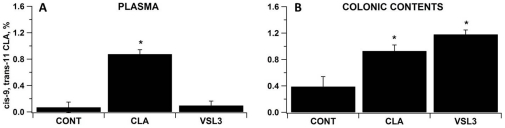
VSL#3 and conjugated linoleic acid (CLA) regulate plasma and colonic CLA concentrations. Cis-9, trans-11 CLA concentrations were measured in plasma (A) and colonic contents (B) by gas chromatography. Values are means ± SEM, n = 8. Statistically significant differences (*P*<0.05) when compared to control (CONT) mice are depicted with an asterisk.

Fatty acid analyses of plasma and colonic content specimens showed an increase in the percentages of cis-9, trans-11 CLA in colonic contents for both CLA and VSL#3 treated mice (Figure 6). However, CLA levels were only increased in the plasma of those mice fed the CLA-supplemented diet (Figure 6), suggesting that the CLA is produced locally by gut microbes in colons of VSL#3-treated mice but cannot be absorbed or distributed systemically.

## Discussion

Manipulation of gut microflora with probiotic bacteria can regulate gut homeostasis and barrier function in part through production of bacterial metabolites [31]. While SCFA are well-established bacterial derived metabolites with effector functions in the gut mucosa, probiotic bacteria can also produce longer chain fatty acids, including CLA, a compound that suppresses inflammation by activating PPAR γ [32]. CLA ameliorates experimental IBD in mice and pigs and CD in humans [2], [4], [11], [33], and other PPAR γ agonists have shown clinical efficacy against human UC [34], [35]. Interestingly, VSL#3 also exerts protective effects in patients with ulcerative colitis [16], [17], [18]. We investigated whether VSL#3 suppresses intestinal inflammation by altering colonic microbial diversity and enhancing microbial CLA production locally that in turn activates PPAR γ in macrophages.

Mice treated with VSL#3 or CLA showed improved DSS colitis that was associated with lower percentages of inflammatory macrophages expressing CCR2 (the receptor for MCP-1) and TNF-α in MLN. Also, both CLA and VSL#3 suppressed colonic mRNA expression of TNF-α and MCP-1, indicating that the protective effect against DSS colitis may be due to a decrease in macrophage recruitment. Of note, dietary CLA recapitulated the effects of VSL#3 administration in mice with DSS colitis, suggesting similarities in the mechanism of action underlying the anti-inflammatory efficacy of CLA and probiotic bacteria.

The ability of VSL#3 and CLA to modulate immune responses and decrease inflammation was assessed by examining the distribution of immune cell subsets at the gut mucosa and systemically. Macrophages of VSL#3-treated mice expressed lower levels of CD11c, a pro-inflammatory cell surface marker implicated in monocyte adhesion to inflamed endothelial cells by binding vascular cell adhesion molecule 1, an activation marker for monocytes/macrophages [36]. The percentages of CD44-expressing monocytes in blood were also lower in PPAR γ-expressing mice treated with VSL#3 when compared to the macrophage-specific PPAR γ null mice, suggesting that VSL#3 reduces colonic inflammation by suppressing the recruitment of F4/80^+^CD11b^+^ monocytes into the colonic LP through a mechanism that is dependent on the expression of PPAR γ in myeloid cells. Moreover, VSL#3 and CLA treatments were associated with a decrease in MCP-1-expressing macrophages at the colonic LP, indicating suppressed inflammatory capacity or decreased number of M1 or classically activated macrophages. Oral administration of either VSL#3 or CLA has been reported to exert dichotomous properties ranging from anti-inflammatory to immunostimulatory. For instance CLA can prevent colitis [2] while enhancing antigen-specific responses to bacterial antigens [37]. VSL#3 can also prevent colitis [20] while enhancing innate immunity at the epithelial barrier level [28]. The role of these interventions as immunostimulatory or immunosuppressive may ultimately depend on other systemic and mucosal factors. Understanding these systems level interactions will help provide a comprehensive mechanistic understanding of their beneficial effects in the gut.

Microbial diversity analyses indicate that both VSL#3 and CLA treatment decreased colonic bacterial diversity in comparison to untreated control mice. VSL#3- and CLA-treated mice showed the lowest diversity of gut bacterial population compared to control untreated mice. In addition, the suppressed colonic bacterial diversity observed in VSL#3 and CLA-treated mice highly correlated with lower histological lesion scores. Our findings coincide with the results of a previous report demonstrating that the probiotic VSL#3 alters the composition of the intestinal microbiota and these changes correlate with disease protection [29]. Additionally, we demonstrate for the first time that CLA treatment can modulate gut microbial diversity.

Plasma metabolomic profiles of VSL#3-treated mice challenged with DSS resembled more closely those of healthy mice than control mice with DSS. These findings are in line with results of a recent study showing that probiotics might correct inflammation-driven metabolic dysfunction [38]. The ability of VSL#3 to produce CLA *in vitro* has been shown in previous studies [39]. We provide evidence that VSL#3 treatment results in high colonic concentrations of cis-9, trans-11 CLA. Interestingly, in contrast to dietary CLA that results in increased plasma levels of both cis-9, trans-11 and trans-10, cis-12 CLA, the plasma cis-9, trans-11 CLA concentrations in VSL#3-treated mice where not different than in control mice, indicating a local effect of microbial-derived CLA without systemic distribution. This local effect may be explained by the limited absorption of long-chain fatty acids at the colonic level. While dietary CLA can be absorbed in the small intestinal and enter the plasma pools, CLA produced by the microbiota is not being absorbed in the large intestine but exerts local immune modulatory and protective effects.

**Figure 7:**
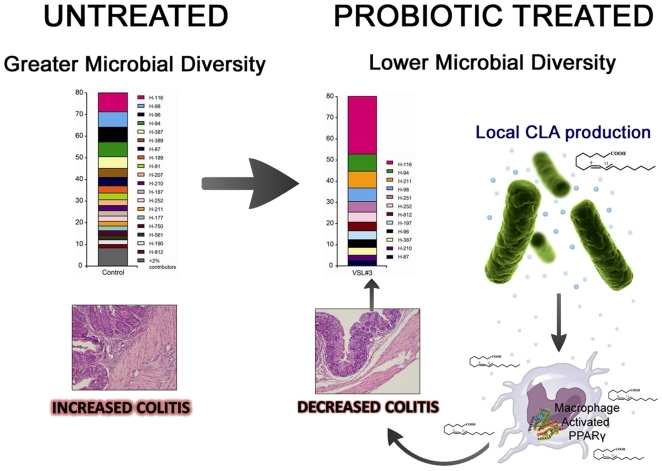
Proposed model for a mechanism of action underlying the protective effects of VSL#3 probiotic bacteria in mouse models of gut inflammation and cancer. Colonization with VSL#3 probiotic bacteria modulate gut microbial diversity and favor local production of conjugated linoleic acid (CLA) in the colon that targets myeloid cell peroxisome proliferator-activated receptor γ (PPAR γ) to suppress colitis.

In conclusion, we provide novel *in vivo* evidence that changes in microbial diversity and local CLA production are implicated in PPAR γ-dependent mechanisms of action underlying the anti-inflammatory and anti-carcinogenic effects of probiotic bacteria (Figure 7). This novel mechanistic model is supported by: results of loss-of-function analyses illustrating the requirement of macrophage PPAR γ in mediating the full spectrum of anti-inflammatory effects of probiotic bacteria in the gut; *in vivo* evidence indicating a reduction of colonic bacterial diversity with a marked predominance of TRF H-116 and local CLA production in colons of VSL#3-treated mice; and remarkable similarities in the ability of probiotic bacteria and CLA to modulate macrophage function at the gut mucosa.

## Supporting Information

Figure S1 **VSL#3 and conjugated linoleic acid (CLA) regulate the plasma metabolome and fecal CLA concentrations.** Histogram demonstrating changes in O-PLS-DA coefficients of individual metabolites related to a dextran sodium sulfate (DSS) challenge in mice administered control (green), CLA (red) and VSL#3 (blue) treatments (A). Positive bars illustrate which metabolites were more abundant in DSS challenge mice; negative ones demonstrate metabolites more abundant in mice without DSS challenge. Confidence intervals derived from jack knifing which do not cross zero line mean that metabolite concentration changes are statistically significant (*P*<0.05).
(DOCX)

Table S1 **Composition of experimental diets.**
(DOCX)

Table S2 **Model summary statistics of metabolic changes in mice plasma following DSS challenge.**
(DOCX)
