# Correction: Probiotic Bacteria Produce Conjugated Linoleic Acid Locally in the Gut That Targets Macrophage PPAR γ to Suppress Colitis

**DOI:** 10.1371/journal.pone.0275513

**Published:** 2022-09-27

**Authors:** The PLOS ONE Editors

The Competing Interests statement for this article [1] is incomplete. Based on information provided in editorial follow up, an updated Competing Interests statement is as follows: At the time of publication of this study, the probiotic formulation sold as VSL#3 was produced by Dupont/Danisco in the US. Claudio De Simone (CDS) is the inventor of this probiotic formulation (also known as the De Simone Formulation). CDS is named as an inventor and assignee on patent “Dietary and pharmaceutical compositions containing lyophilized lactic bacteria, their preparation and use” (U.S. Patent No. 5,716,615) and the sole owner of the know-how used to formulate the product. At the time of publication of the article, CDS owned one share of stock in, and served as Chief Executive Officer and Director for, VSL Pharmaceuticals, Inc., which sold the De Simone Formulation until 2016. At the time of publication of this study, co-author Josep Bassaganya-Riera was a *PLOS ONE* Editorial Board member. This does not alter the authors’ adherence to all the *PLOS ONE* policies on sharing data and materials.

The VSL#3 used in this study was obtained from VSL Pharmaceuticals and produced by Danisco/Dupont in the US.
