# Supplementary material for: Probiotic Bacteria Produce Conjugated Linoleic Acid Locally in the Gut That Targets Macrophage PPAR γ to Suppress Colitis
Source: PLoS One. 2012 Feb 21;7(2):e31238. doi: 10.1371/journal.pone.0031238 (PMC3283634; doi:10.1371/journal.pone.0031238)
Supplement: Figure S1 — VSL#3 and conjugated linoleic acid (CLA) regulate the plasma metabolome and fecal CLA concentrations. Histogram demonstrating changes in O-PLS-DA coefficients of individual metabolites related to a dextran sodium sulfate (DSS) challenge in mice administered control (green), CLA (red) and VSL#3 (blue) treatments (A). Positive bars illustrate which metabolites were more abundant in DSS challenge mice; negative ones demonstrate metabolites more abundant in mice without DSS challenge. Confidence intervals derived from jack knifing which do not cross zero line mean that metabolite concentration changes are statistically significant (P<0.05). (DOCX) [file pone.0031238.s001.docx]

**Supplementary Figure S1**

**
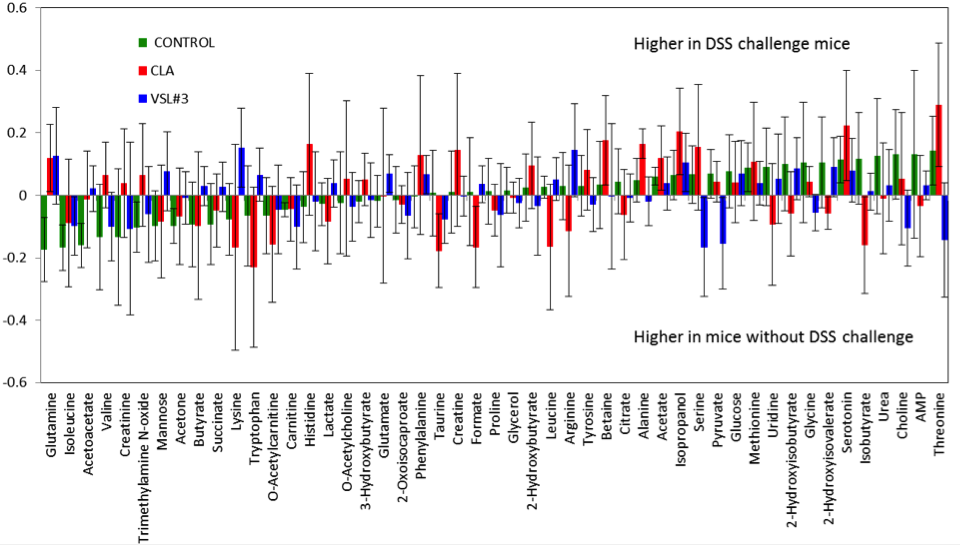
**

**Supplementary Figure S1. VSL#3 and conjugated linoleic acid (CLA) regulate the plasma metabolome and fecal CLA concentrations.** Histogram demonstrating changes in O-PLS-DA coefficients of individual metabolites related to a dextran sodium sulfate (DSS) challenge in mice administered control (green), CLA (red) and VSL#3 (blue) treatments (A). Positive bars illustrate which metabolites were more abundant in DSS challenge mice; negative ones demonstrate metabolites more abundant in mice without DSS challenge. Confidence intervals derived from jack knifing which do not cross zero line mean that metabolite concentration changes are statistically significant (*P* < 0.05).
