# Supplementary material for: Probiotic Bacteria Produce Conjugated Linoleic Acid Locally in the Gut That Targets Macrophage PPAR γ to Suppress Colitis
Source: PLoS One. 2012 Feb 21;7(2):e31238. doi: 10.1371/journal.pone.0031238 (PMC3283634; doi:10.1371/journal.pone.0031238)
Supplement: Table S2 — Model summary statistics of metabolic changes in mice plasma following DSS challenge. (DOCX) [file pone.0031238.s003.docx]

**Supplementary Table S2.** Model summary statistics of metabolic changes in mice plasma following DSS challenge.

| **Diet** | **R^2^X** | **R^2^Y** | **Q^2^Y** |
| --- | --- | --- | --- |
| **Control** | 0.343 | 0.973 | 0.519 |
| **CLA** | 0.609 | 0.998 | 0.428 |
| **VSL#3** | 0.197 | 0.884 | 0.0472 |

Explanation: R^2^X – explained variance of X, metabolite concentration dataset; R^2^Y – explained variance of Y, DSS challenge status of samples; Q^2^Y – predicted variance of Y estimated using cross-validation.

**Metabolomic analyses of plasma samples**

Blood samples were collected from each mouse post-mortem during the necropsy. Blood samples were then centrifuged at 5000 rpm at room temperature for 10 minutes to plasma collection and frozen. Plasma samples were thawed and filtered using 3-kDa Nanosep microcentrifuge filters to remove proteins. Samples were brought to 400 μl by addition of D20, 80 μl of phosphate buffer containing dimethyl-silapentane-sulphonate (final concentration 0.5 mM) and 10 μl of 1M sodium azide. The final sample pH was adjusted to 7 ± 0.01. All NMR experiments were performed on a Bruker Avance 600 spectrometer (Bruker BioSpin Ltd., EastMilton, ON, Canada) operating at 600.22 MHz and equipped with a 5-mm TXI probe at 298 K. Regular one-dimensional proton NMR spectra were acquired using a standard Bruker pulse sequence (prnoesy1d) in which the residual water peak was irradiated during the relaxation delay of 1.0 second and during the mixing time of 100 millisecond. A total of 1,024 scans were collected into 63,536 data points over a spectral width of 12,195 Hz with a 90-degree pulse width and a 5-second repetition time. A line broadening of 0.2 Hz was applied to the spectra prior to Fourier transformation, phasing, and baseline correction. Additional 2-dimensional NMR experiments were performed for the purpose of confirming chemical shift assignments, including homonuclear total correlation spectroscopy (2D ^1^H-^1^H TOCSY) and heteronuclear single quantum coherence spectroscopy (2D ^1^H-^13^C HSQC), using standard Bruker pulse programs. Forty nine metabolites were assigned based on comparison of both ^1^H and ^13^C chemical shifts and spin-spin coupling constants with those of model compounds in Human Metabolome Database (HMDB)^2^ and Chenomx NMR Suite 7.0 software (Chenomx Inc., Edmonton, Canada). Metabolites were quantified using the targeted profiling approach as implemented in the Chenomx software ^3^. The metabolite concentration data matrix was analyzed by pattern recognition methods within SIMCA-P (version 12.0, Umetrics). A supervised O-PLS-DA approach was chosen as previously described ^4^.

**References**

1. Evans NP, Misyak SA, Schmelz EM, Guri AJ, Hontecillas R, Bassaganya-Riera J. Conjugated linoleic acid ameliorates inflammation-induced colorectal cancer in mice through activation of PPARgamma. J Nutr 2010;140:515-21.

2. Wishart DS, Knox C, Guo AC, Eisner R, Young N, Gautam B, Hau DD, Psychogios N, Dong E, Bouatra S, Mandal R, Sinelnikov I, Xia J, Jia L, Cruz JA, Lim E, Sobsey CA, Shrivastava S, Huang P, Liu P, Fang L, Peng J, Fradette R, Cheng D, Tzur D, Clements M, Lewis A, De Souza A, Zuniga A, Dawe M, Xiong Y, Clive D, Greiner R, Nazyrova A, Shaykhutdinov R, Li L, Vogel HJ, Forsythe I. HMDB: a knowledgebase for the human metabolome. Nucleic Acids Res 2009;37:D603-10.

3. Weijie AM, Newton J, Mercier P, Carlson E, Slupsky CM. Targeted profiling: quantitative analysis of 1H NMR metabolomics data. Anal Chem 2006:4430-4442.

4. Trygg J, Holmes E, Lundstedt T. Chemometrics in metabonomics. J Proteome Res 2007;6:469-79.
